# Modern Sociology

**Published:** 1899-02-25

**Authors:** 


### MODERN SOCIOLOGY.

### WOMEN AS SANITARY INSPECTORS.

AMONG the many occupations that have been opened for women of late years, that of sanitary inspector is as yet one of the least known, and therefore one of the least popular. At the first blush it seems one with which one does not like to associate the gentler and most fastidious sex; but, after all, the work has, for the most part, to do with dwelling-houses, where women rule supreme, and where a female inspector would meet with less opposition than a man, whom the matriarch of the tenement house looks upon as an inferior being, with no ideas worth considering on domestic matters. There a lady inspector, armed with technical knowledge and with the might of the municipality behind her, might do incalculable good. As a matter of fact women inspectors are employed in many places, and where once employed they have never been dismissed.

It is twenty-five years since the chief sanitary inspector of Glasgow began to employ women among his subordinates in the poorer parts of the town. Till 1892, however, the Scottish city had no imitator. But the passing of the Factory and Workshops Act in 1891, which placed these places under the supervision of local authorities, drew the attention of these local authorities to the difficulties with which sanitary inspectors had to contend in dealing with workshops where women were chiefly, or exclusively, employed, and the improvement which might be effected by employing women as coadjutors or substitutes for men. Under these circumstances, the town of Nottingham appointed Miss Hawberry as workroom inspector in 1892. In the following year Manchester and Kensington appointed women as inspectors, but only, as in Nottingham, as inspectors of workrooms. The inspectors had to state in their reports the description of trade or business carried on in the shop they were reporting on; the dimensions (length, height, breadth, and cubic space) of each workroom, the number of gas-burners in each, the maximum number of occupiers allowed, the means of ventilation, the number and position of sanitary conveniences, and any other remarks they thought important. In another book were kept the details of the conditions found on each visit, such as the number of persons actually found in each workroom, the state of the room as regards warmth, the means of heating, the condition of the premises, including the condition of the sanitary conveniences, the condition and situation of the dust-bin, the supply of drinking water, and the like. On another form they reported overcrowding or other forms of nuisance.

The example thus set has been followed in several districts of London, as well as in Manchester, Liverpool, Leicester, Brighton, and St. Helens. Some of the ladies were appointed in the first place as workroom inspectors only, but in most they are now employed to investigate tenement houses also. They report as to the number of families in their district who are found to be cleanly in their house and habits, and those who are the reverse. It is possible that in this matter of domestic cleanliness the woman-inspector may be more fastidious than her male colleague. It is to be hoped, therefore, that a sanitary inspector of the housekeeping sex will help, by her recommendations and reproofs, to raise the standard of cleanliness among the occupants of the tenements. It is to be hoped, however, that in the process she may not destroy that self-respect which is the best lever to raise their condition, both moral and social. To succeed in her work the woman-inspector must have endless patience, tact, and good sense.

But, given these essentials, the housewifely knowledge that a woman of the middle classes generally has may be of endless use to the poorer women whom she meets in the course of her work. Even in condemning a choked drain it is easy to give a hint as to the treatment drains require in order to prevent such accidents. Suggestions as to management, cookery, the feeding and clothing of children, may not belong, strictly speaking, to sanitary science, or at least to the work of a sanitary inspector, but if they were given in a kindly spirit, and acted upon, they would do much to destroy those circumstances which cause the visits of a sanitary inspector to be necessary. We do not mean to imply that such domestic knowledge is the sole requisite of the woman inspector. On the contrary, it would be a mistake to appoint anyone who had not undergone a course of public health training, both theoretic and practical. We would not recommend the appointment of a woman on the ground that a woman, being imperfectly trained, can be got cheaper than a man. The salary is a matter for every district to decide for itself and its servants; but the quality of the worker ought not to be a matter for question. Thorough knowledge of the work she has undertaken is an essential of the lady inspector's equipment, as of her male colleague's, and the appointment of women should not mean the appointment of an inferior class of investigators. But, given the proper training, those feminine qualities we have touched on might be added with advantage to the investigation and the investigated. The remarks of a journalist who accompanied the lady inspector employed by the parish of St. Pancras are worth quoting in this connexion. After describing the details of a day's work—the visits to rag-shops, laundries, and flower factories—she concludes: "The tact wherewith the inspector pursued her calling was admirable. To a novice it appeared somewhat appalling to invade a front door, demand a sight of the dust-bin, unravel the mysteries of back-yards, and trace the source of drinking water. But the inquisitional nature of our inquiries was conducted most delicately by the sanitary lady, whose visiting-card set forth her authority to inspect workshops. She invariably remembered that on the occasion of a previous visit the baby was ill, or the husband laid up, and her inquiries on these points established a kindly sympathy which softens the nature of the poor. As one of the women put it: 'If we must have somebody ferretin' in our dust-bins and back-yards we'd rather have a lady; and,' she added, 'she is a nice young lady, too.'" To gain such relations with one's clients—to call them by their name—is the first step to persuading them to adopt your recommendations; and it is because women of the right stamp can do this that we recommend their still wider employment as colleagues and assistants to male sanitary inspectors.

